# Special consideration in the dosing of medications for patients with COVID-19 and acute kidney injury

**DOI:** 10.1590/S1677-5538.IBJU.2020.1039

**Published:** 2020-12-30

**Authors:** Chia Siang Kow, Syed Shahzad Hasan

**Affiliations:** 1 International Medical University School of Postgraduate Studies Kuala Lumpur Malaysia School of Postgraduate Studies, International Medical University, Kuala Lumpur, Malaysia.; 2 University of Huddersfield Department of Pharmacy Huddersfield United Kingdom Department of Pharmacy, University of Huddersfield, Huddersfield, United Kingdom.; 3 University of Newcastle School of Biomedical Sciences & Pharmacy Callaghan Australia School of Biomedical Sciences & Pharmacy, University of Newcastle, Callaghan, Australia.

*To the editor*,

The review by Gotera (1) that summarized evidences surrounding the available therapeutic agents in patients with coronavirus disease 2019 (COVID-19) is commendable. In addition, the authors listed the possible side effects associated with each of these therapeutic agents, which could serve as a reminder for clinicians who may not be familiar with the safety profile of new or existing but repurposed therapeutic agents for the treatment of COVID-19.

We read with interest the authors’ recommendation of prescribing and adjusting the dosage of therapeutic agents in patients with COVID-19 and concurrent renal impairment (1). Although the recommended dosage adjustment of a few COVID-19-specific pharmacological treatments had been based on clear cutoffs of renal function, we afraid that the recommended approach may be misleading, specifically for patients with COVID-19 and concurrent acute kidney injury.

Up to 78% of hospitalized patients with COVID-19 developed acute kidney injury (2). It should be noted that the measured serum creatinine in patients who develop acute kidney injury may not correspond accurately to the true glomerular filtration rate since the serum creatinine in this patient population is usually not in a steady-state (3). If the serum creatinine is actively increasing, the glomerular filtration rate estimated based on the measured serum creatinine is likely being overestimated. On the other hand, if the serum creatinine is actively falling, the glomerular filtration rate estimated on the basis of the measured serum creatinine is likely being underestimated.

Since the authors only specified in the table that a given therapeutic agent should be avoided or prescribed with dose adjustment if less than certain threshold creatinine clearance, it may lead to misprescribing or potential omission in patients with COVID-19 associated acute kidney injury. To illustrate, remdesivir that should be avoided in patients with creatinine clearance of <30 mL/min/1.73m^2^ as specified, can be mistakenly prescribed to patients with COVID-19 associated acute kidney injury in the settings of actively rising serum creatinine, and whose glomerular filtration rate estimated on the basis of the measured serum creatinine is or slightly more than 30 mL/min/1.73m2, where in fact, the glomerular filtration rate is likely being overestimated. On the other hand, remdesivir can be mistakenly omitted in patients with COVID-19 associated acute kidney injury in the settings of actively decreasing serum creatinine, whose creatinine clearance estimated based on the measured serum creatinine is slightly less than 30 mL/min/1.73m^2^, where in fact, the glomerular filtration rate is likely being underestimated.

In order to address this non-steady-state phenomenon of serum creatinine in patients with acute kidney injury, some have proposed a kinetic glomerular filtration rate formula, though such formula had yet to be clinically validated (4). Therefore, the following caveats can be specifically followed for patients with COVID-19 associated acute kidney injury:

1. If the serum creatinine is actively falling, the medications should be dosed according to a creatinine clearance higher than that estimated with the measured serum creatinine.
2. If the serum creatinine is actively increasing (or if only a single initial measurement is available), the creatinine clerance should be presumed to be 0 mL/min, and the dose of drugs should be adjusted accordingly (or the drug should be omitted altogether).
  The Authors
